# Medical prescription and nursing administration of medication in learning disabilities in-patient settings

**DOI:** 10.1192/bjo.2021.942

**Published:** 2021-06-18

**Authors:** Ellen Williams, Ansar Choudry, Kabeer Hussain, Praveen Ravi, Sadia Zahid

**Affiliations:** Black Country Partnership NHS Foundation Trust

## Abstract

**Aims:**

The aim of this re-audit was to review whether inpatientprescription cards are completed correctly by doctors and administered by nurses, and to compare the results with the previous audit.

**Background:**

We carried out a re-audit of Medical Prescription and Nursing Administration of Medication in Learning Disabilities In-patient Settings. Black Country Partnership NHS Foundation Trust is committed to managing medicines safely, efficiently and effectively as a key part of delivering high quality patient centred care. In BCPFT medications are recorded by doctors on paper prescription cards and administered by registered nurses.

**Method:**

This audit compared results against the standards for prescribing medication in BCPFT Medicines Policy.Prescription charts were retrospectively reviewed against 22 standards for all LD inpatients as outlined in the LD trust policy across all 3 of the Learning Disabilities in-patient units during May 2019 as long as they were still inpatients during this month. 27 prescription cards were reviewed in total.

**Result:**

100% of prescription cards had patients full names , address , ward name, were fully legible , written in black ink, route of administration, approved abbreviation for route, date of prescription, signature of prescriber , prescription labelled as 1of 1 /2, frequency of prn meds and indication . Whereas only 96% had generic drug names, clearly documented doses and time of administration along with acceptable abbreviation and appropriate code for omission. 85% drugs had a stop date once drug was stopped and 85% had allergies recorded in red and had a line drawn through once drug was omitted.

**Conclusion:**

The re- audit was highlighted to inpatient managers, nursing staff, The Medicines Management Committee (MMC) anddoctors in the Learning Disability division. Prescribers werereminded of the importance of documenting a stop date for the prescriptions and signing off once drug is crossed out. It was discussed in MMC to consider removing the standard for recording allergies in red ink as the box is already red in colour. The PRN section for medication does not have an area to sign when the drug is cancelled and this in particular is the case when PRN medication is re-written. It was discussed to limit this standard to regular medication and to be taken in consideration if the current drug chart requires redesigning in the future. We also recommended a re- audit in 2 years’ time.

